# Supplementary material for: Incidental Indeterminate Renal Lesions: Distinguishing Non-Enhancing from Potential Enhancing Renal Lesions Using Iodine Quantification on Portal Venous Dual-Layer Spectral CT
Source: J Pers Med. 2023 Oct 28;13(11):1546. doi: 10.3390/jpm13111546 (PMC10672440; doi:10.3390/jpm13111546)
Supplement: Supplementary file 1 [file jpm-13-01546-s001.zip › Supplemental figure S1.pdf]

Supplemental figure S1. Box and whisker plot showing the relationship between enhancement on multiphase CT and iodine concentration for simple cysts (0-19 HU), hyperattenuating cysts (≥70 HU) and potential renal masses (20-69 HU) based on true unenhanced CT.

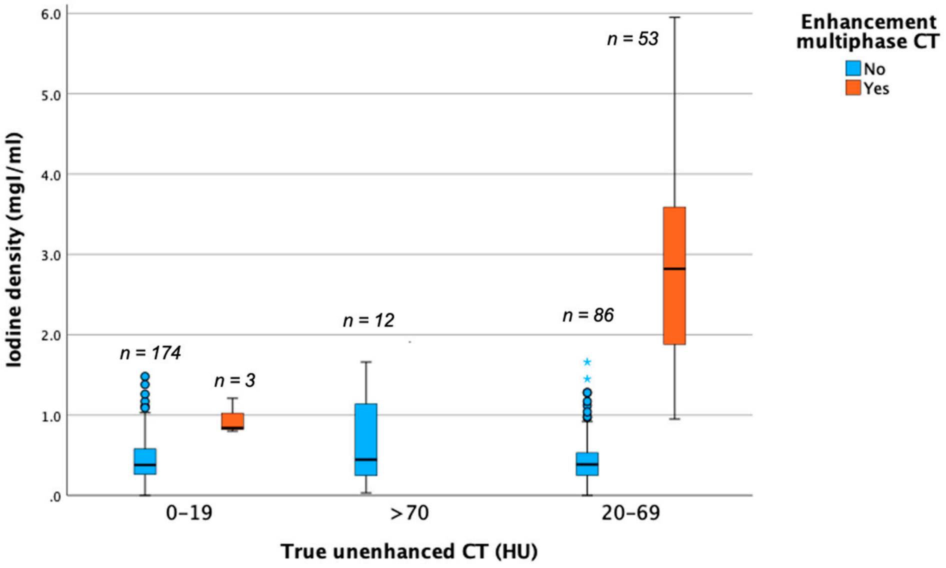

Enhancement: ≥20 ΔHU between true unenhanced and portal venous phase CT
Horizontal lines in box represents median value. Top and bottom of boxes represent 25th–75th percentiles of data values. The whiskers (vertical lines) represent minimum and maximum values (excluding outliers).

- *Outlier: 3rd quartile + 1.5*interquartile range or 1st quartile – 1.5*interquartile range*

* *Extreme outier: 3rd quartile + 3*interquartile range or 1st quartile - 3*interquartile range*
