# Supplementary material for: Incidental Indeterminate Renal Lesions: Distinguishing Non-Enhancing from Potential Enhancing Renal Lesions Using Iodine Quantification on Portal Venous Dual-Layer Spectral CT
Source: J Pers Med. 2023 Oct 28;13(11):1546. doi: 10.3390/jpm13111546 (PMC10672440; doi:10.3390/jpm13111546)
Supplement: Supplementary file 1 [file jpm-13-01546-s001.zip › Supplemental figure S2.pdf]

**Supplemental figure S2.** Box and whisker plot showing the relationship between quantity of enhancement on multiphase CT and iodine concentration.

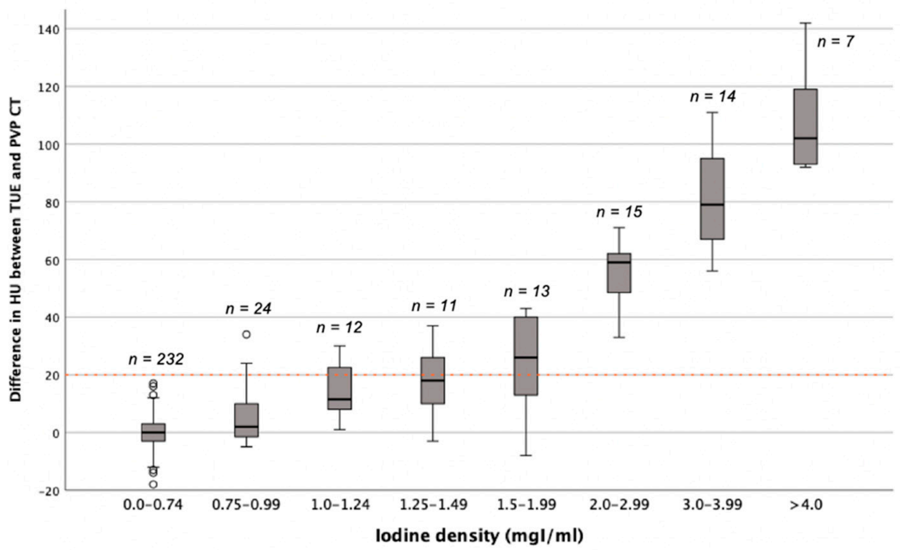


*TUE: true unenhanced; PVP: portal venous phase*

*Red dotted line: enhancement on multiphase CT (≥20 ΔHU)*

*Horizontal lines in box represents median value. Top and bottom of boxes represent 25th–75th percentiles of data values. The whiskers (vertical lines) represent minimum and maximum values (excluding outliers).*

o *Outlier: 3rd quartile + 1.5*interquartile range or 1st quartile – 1.5*interquartile range*
